# Anxiety and COVID-19 Anxiety in Positive Youth Development: A Latent Profile Analysis Study

**DOI:** 10.1007/s10964-023-01829-z

**Published:** 2023-07-27

**Authors:** Tina Pivec, Ana Kozina

**Affiliations:** Educational Research Institute, Ljubljana, Slovenia

**Keywords:** Positive Youth Development, The Five Cs, Anxiety, COVID-19 anxiety, Latent profile analysis

## Abstract

The COVID-19 pandemic has accelerated anxiety and triggered a new and specific COVID-19 anxiety in all age groups, especially in adolescence. The aim of the present study was to identify differences between anxiety and COVID-19 anxiety in profiles of positive outcomes by using the Positive Youth Development (PYD) framework (i.e., the Five Cs of competence, confidence, character, caring, and connection) on a sample of 1,977 adolescents from Slovenia (57.4% females; *M*_*age*_ = 15.34 years) from lower- and upper-secondary schools. Latent profile analysis revealed four distinctive profiles: High PYD, Self-efficacious, Socio-emotional, and Low PYD. The High PYD profile reported lower anxiety and moderate COVID-19 anxiety; the Self-efficacious profile reported lower anxiety and the lowest COVID-19 anxiety; the Socio-emotional profile reported moderate anxiety and the highest COVID-19 anxiety; and the Low PYD profile reported higher anxiety and moderate COVID-19 anxiety. The study findings suggest that higher levels of competence, confidence, and connection can mitigate anxiety, while higher levels of caring are associated with COVID-19 anxiety. Nonetheless, the study supports the promotion of all Five Cs of the PYD framework to prevent anxiety, COVID-19 anxiety, and maladaptive developmental outcomes. The study provides important insights into profiling responses to extreme situations, such as the COVID-19 pandemic.

## Introduction

There are two critical points of concern with regard to the association between adolescence and anxiety in the current COVID-19 pandemic. Firstly, adolescence is a crucial developmental stage wherein the onset of anxiety disorders can manifest (Kessler et al., 2005). Secondly, the pandemic has led to the closure of schools, increased social isolation, and loneliness, all of which have further contributed to the rise in anxiety among adolescents (Racine et al., 2021). As potential protective factors, the Five Cs of Positive Youth Development (PYD) have been identified (Holsen et al., 2017). They consist of competence, confidence, character, caring, and connection and can serve as fundamental building blocks for the development of effective prevention and intervention measures, particularly in times of extreme crisis. Additionally, the adoption of a person-centered approach can aid in the identification of vulnerable groups of adolescents in need of interventions, thereby enabling the design of targeted prevention and intervention measures (Geldhof et al., 2019). Research that adopted a person-centered approach while incorporating the Five Cs is scarce, even though it is crucial to examine how the distribution of the Five Cs differs among the profiles of adolescents in order to investigate how profiles are linked to several positive and negative outcomes (e.g., internalizing and externalizing behavior). These findings can help understand specific developmental regulations and facilitate the development of more effective and targeted anxiety prevention and intervention programs, particularly in times of increased risk such as the COVID-19 pandemic. Thus, the present study has two objectives. Firstly, it aims to employ a person-centered approach to identify distinct profiles among adolescents by analyzing the distribution of the Five Cs of PYD, with age and gender being considered as covariates in the analysis. Secondly, it aims to investigate the associations between these identified profiles and both general anxiety and situation-specific COVID-19 anxiety.

### Anxiety and COVID-19 Anxiety in Times of COVID-19 Pandemic

Anxiety is one of the most frequent psychological challenges in adolescence (Neil & Christensen, 2009) and has been on the increase in recent decades (Kozina, 2014). It is a natural reaction to a perceived threat that is manifested through cognitive (e.g., worries, racing thoughts), physiological (e.g., autonomic arousal), and behavioral (e.g., escape, avoidance) responses (Abramowitz & Blakey, 2020). Moreover, in addition to higher levels of anxiety during the COVID-19 pandemic (Erbiçer et al., 2022), situation-specific COVID-19 anxiety has also emerged. It involves fears and worries associated with the COVID-19 pandemic (e.g., concerns about getting infected, concerns about the uncertainty of the future, concerns about the health of important others, and concerns about the social consequences of the pandemic). While anxiety is a tendency to react anxiously in various life situations (trait anxiety), COVID-19 anxiety is a highly specific situational anxiety that has arisen during the COVID-19 pandemic, and consequently, different patterns of responses can occur for each type of anxiety.

Several triggers might explain the increase in anxiety during a pandemic, such as the presence of a threat, an uncertain future, social isolation, and loneliness (Racine et al., 2021). First of all, the presence of a real health threat in the form of the COVID-19 virus itself could be a major contributor to COVID-19 anxiety symptoms. Additionally, the uncertainty about the future, including unknowns such as when the pandemic will end, when schools will reopen, and how final exams will be conducted, can also contribute to maintaining or exacerbating anxiety symptoms (Jacoby, 2020). Another significant trigger of anxiety during the pandemic has been social isolation, which has in turn led to increased feelings of loneliness. The longer that isolation has persisted, the greater the risk of anxiety and depressive disorders (Loades et al., 2020). Of all the measures taken to prevent the spread of COVID-19, school closures have had the most significant impact on the lives of youth. Together with other measures, they have led to increased levels of depression and anxiety among students while their positive mental well-being has decreased (Houghton et al., 2022).

Slovenia experienced one of the longest school closures among EU countries during the height of the COVID-19 pandemic in 2020–2021 (UNESCO, n.d.). Our study was conducted before and at the onset of the second school closure in the fall of 2020, a time marked by heightened stress levels. In addition to school closures, the Slovenian government implemented further measures, including restrictions on movement between regions and municipalities, mandatory wearing of masks in public spaces, and limitations on outdoor gatherings to no more than six individuals. As a result, all school and extracurricular activities were either canceled or moved online. Findings from the REDS survey (Klemenčič Mirazchiyski et al., 2021), which assessed the educational disruption caused by the pandemic during the first wave, reveal that 53% of Slovenian youth reported increased levels of loneliness during school closures, while about half of them experienced higher levels of anger than usual. Moreover, 39% had sleeping difficulties compared to before the pandemic, approximately 60% reported worries about changes in schooling and how school closures would impact their learning and future education, and 72% referred to missing their classmates. These findings align with several potential triggers of anxiety, including loneliness, increased concerns about the uncertain future, and social isolation.

### Positive Youth Development

Contextual changes, such as the COVID-19 pandemic, have had a profound impact on youth development. One of the theoretical perspectives that highlight the importance of the relationship between the individual and the contexts is the Positive Youth Development perspective (PYD). In response to the deficit-oriented approaches traditionally used in studying adolescence, PYD incorporated a strengths-based approach to youth development (Lerner et al., 2019). It is embedded in the relational developmental systems metatheory (Overton, 2015), which emphasizes the mutual relations between individuals and their contexts. Thus, in the PYD perspective, youth is studied as the product of an interaction between individual characteristics and youth contextual resources (i.e., school, family, community, and society) – known as “developmental regulations” (Lerner et al., 2005). Developmental regulations are based on the concept of relative plasticity, acknowledging that individuals are active agents in their developmental processes. Developmental changes occur through a reciprocal exchange between the individuals and their surrounding contexts. When there is a harmonious balance between the strengths of the individuals and the supportive nature of their contexts, these developmental regulations can be termed adaptive developmental regulations (Lerner et al., 2019).

Through the dynamic processes of adaptive developmental regulations, PYD outcomes conceptualized as the Five Cs – competence, confidence, character, caring, and connection (Lerner et al., 2005) – can emerge. Competence is a positive aspect of one’s abilities within certain domains (e.g., social and academic skills). Confidence entails an intrinsic sense of positive self-worth and self-efficacy. Character refers to the adherence to standards for appropriate behavior with regard to social and cultural norms and moral integrity. The presence of sympathy and empathy towards others serves as an indicator of caring. Connection represents all positive reciprocal relationships that youth maintain with significant others and institutions (Lerner et al., 2005). While the Five Cs have been primarily studied as a global PYD factor or as distinct components, an alternative two-factor model has been proposed (Årdal et al., 2018). This model combines competence and confidence in so-called “efficacious Cs” and connection, caring, and character in so-called “socioemotional Cs.” Additionally, some gender-specific differences among youth were acknowledged across the Five Cs (Årdal et al., 2018), as females reported higher connection, character, and caring and males reported higher confidence and competence. Regarding age, there is not a clear consensus since previous studies did not detect any age differences across the Five Cs (Gomez‐Baya et al., 2019), or only perceived differences between younger and older adolescents (younger adolescents had higher caring, character, and connection; Conway et al., 2015). There is evidence that the Five Cs are positively related to an adolescent’s contribution (the Sixth C) to him- or herself, to his or her family, and society (Lerner et al., 2005), as well as being negatively related to risky behaviors and emotional difficulties, such as anxiety (Holsen et al., 2017).

Empirical evidence supports the notion that the Five Cs lead to positive outcomes only when they result from the mutually favorable relationships between the individuals and their unique contexts – adaptive developmental regulations. However, it is crucial to acknowledge that the nature of the relationship between an individual and their context is not inherently positive, but can instead be neutral or even negative (Geldhof et al., 2019) since each C of the Five Cs can have a unique association with developmental outcomes. Therefore, when examining the developmental regulations, it is essential to consider that non-adaptive developmental regulations (i.e. neutral or negative developmental regulations) can occur, having a neutral or negative impact on individual’s and contexts’ co-regulation of the development. While neutral developmental regulations have no discernible impact on individuals or their contexts, negative developmental regulations can lead to detrimental effects. Negative developmental regulations can harm the individual (sacrificial developmental regulations, martyring developmental regulations), the context (parasitic developmental regulations), or both (maladaptive developmental regulations) (Geldhof et al., 2019). Sacrificial developmental regulations relate to the changes in the context that can harm the individual but benefit the context (e.g., closing schools during the pandemic, which helped to control the spread of the disease but had a negative impact on youth) while martyring developmental regulations include undertaking self-sacrificing actions at a personal cost (i.e., having higher character, caring, and inadequate competence and confidence may cause these developmental regulations since these individuals tend to prioritize helping others and caring for others while neglecting their own well-being). Further, parasitic developmental regulations benefit the individual and harm their context (i.e., individuals with high competence, confidence, and low caring and character may have the potential to harm their context to fulfill their desires) (Geldhof et al., 2019). Lastly, maladaptive developmental regulations relate to harming both the individual and the context (i.e., having low levels of the Five Cs can lead to several negative outcomes, including emotional difficulties and risky behaviors; Jelicic et al., 2007). Nevertheless, depending on relative plasticity these developmental regulations may change to adaptive ones and, thus, lead to a more optimal development (Lerner et al., 2019).

One promising approach to gaining a deeper understanding of the relationship between the Five Cs and maladaptive outcomes is through the application of person-centered analysis. Person-centered analysis enables researchers to identify subgroups based on their responses, thereby classifying similar participants into distinct classes or profiles (Nylund-Gibson & Choi, 2018). Research on PYD profiles based solely on the Five Cs is very scarce as, to the best of our knowledge, only three studies have included the Five Cs as indicators in the person-centered approach (i.e., latent class analysis or latent profile analysis). All three studies confirmed the notion that several different profiles could be identified. In a longitudinal study (Johnson, 2021), the profiles in the first wave were determined by the presence and strength of each of the Cs, with the following profiles being identified: high overall, moderate with caring emphasis, midpoint, midpoint with caring emphasis, and moderately high overall. In the second wave, the profiles were similar to the first one, except for the low moderate with caring emphasis profile emerging and the midpoint profile disappearing. Additionally, the size of the profiles varied in each wave, although the moderate with caring emphasis, high overall, and moderate overall profiles remained stable in size across both waves. Another study (Ferrer-Wreder et al., 2021) found two profiles, with one having lower caring and character scores than the other, but with the two of them being similar in terms of competence, confidence, and connection. The profile with higher caring and character scores reported more somatic complaints and higher general and social well-being. Furthermore, profiles of the Five Cs in four different data sets and their relation to contribution were also examined (Johnson & Ettekal, 2023). While each latent profile analysis revealed a different number of profiles, some similarities were visible among them. Specifically, in all data sets, the profile with low caring scores emerged, while profiles with high scores on character and caring were the largest in almost all data sets. Connection did not differ between profiles. Further, profiles with higher scores on all of the Five Cs tended to have a higher contribution. Additionally, some studies (e.g., Nylund-Gibson & Choi, 2018) were based on the PYD but employed different measures or used the Five Cs as covariates in the analysis (e.g., Arbeit et al., 2014). However, to the best of our knowledge, none of the aforementioned studies was conducted during the COVID-19 pandemic.

### Positive Youth Development in Times of COVID-19 Pandemic

Amidst the COVID-19 pandemic, immersing changes in youth’s life occurred that had an impact both on the adolescents and their contexts. PYD attributes were found to serve as protective factors in reducing the negative influence of traumatic situations (e.g., the COVID-19 pandemic) on adolescent mental health (Shek et al., 2021). The presence of PYD attributes within individuals can serve as a buffer against the adverse psychological consequences associated with such challenging circumstances. It is also worth noting that several studies (see Ma et al., 2021 for review) focused on the impact of the pandemic on mental health and its risk and protective factors. These factors, which are in line with certain aspects of PYD, encompass elements such as positive family relationships and social support. However, only a limited number of studies have investigated the changes in the Five Cs during the COVID-19 pandemic, with only one study comparing PYD indicators before and during the pandemic. This study revealed a decrease in general PYD and total PYD scores (assessed by the Chinese PYD Scale) after the pandemic has started (Wang et al., 2023). Additional insights come from the Slovenian context as well since connection, caring, and character also decreased during a school year during the COVID-19 pandemic, while competence and confidence increased at the same time (Kozina & Wiium, 2023).

### Anxiety and the Positive Youth Development Perspective

PYD should lead to lower levels of internalizing behaviors (e.g., anxiety, depression; Holsen et al., 2017). However, the relationship between anxiety and the Five Cs is complex and depends on how the Five Cs are treated as indicators of PYD. When a global PYD factor has been examined, studies have found a negative association between the global PYD and anxiety (Geldhof et al., 2014b). However, when the Five Cs were studied as distinct components, not all of them were negatively associated with anxiety (Kozina et al., 2021a). More specifically, research has shown that anxiety is negatively associated with academic competence (Mazzone et al., 2007) and social competence (Settipani & Kendall, 2013). In terms of confidence, self-efficacy is a negative predictor of anxiety, especially the cognitive part of anxiety such as worrying (Tahmassian & Moghadam, 2011); on the other hand, higher self-confidence can help individuals to cope more successfully with day-to-day challenges (Soleimani et al., 2017). Furthermore, connection is important as well since adequate peer support and satisfying friendships can buffer social anxiety (Erath et al., 2010). On the other hand, negative relationships with parents (e.g., parents’ aggressive behavior) contribute to higher anxiety, while there is no connection between positive relationships with parents and anxiety (Schwartz et al., 2012). With regard to the relationship between character and anxiety, research has shown that highly anxious individuals tend to exhibit stricter adherence to rules, greater inhibition, and less impulsivity (Eisenberg et al., 2010). Interestingly, character was a significant positive predictor of anxiety in a sample of Portuguese individuals, but not in samples from Slovenia and Spain (Kozina et al., 2021a). On the other hand, higher levels of caring have been associated with higher levels of anxiety (Holsen et al., 2017). This can be a result of emotional contagion (i.e. not being able to differentiate between one’s emotional state and the emotional state of the other) or empathic over-arousal (i.e. being so overwhelmed by the other person’s feelings that the person focuses on his or her feelings instead of helping the person that was initially under stress; Hoffman, 2008). A person-centered approach may provide additional clarity regarding the complex relationships between the Five Cs and anxiety, as well as establish whether there are differences between anxiety and anxiety specifically related to the COVID-19 pandemic.

## Current Study

Previous studies investigated the relationship between the Five Cs and internalizing or externalizing behavior, however, only a few have captured the nuances of the distribution of each of the Five Cs within individuals. Further, while several studies provided the rationale for including the Five Cs in anxiety prevention, the interplay among the Cs in relation to anxiety has not been well-examined. Moreover, although many studies have highlighted the escalation of anxiety, including situation-specific COVID-19 anxiety, during the COVID-19 pandemic, these phenomena have not been analyzed through the lens of the PYD perspective. In light of these research gaps, the aims of the current study are twofold: firstly, the person-centered approach will be employed to identify the profiles of adolescents by examining the distribution of the Five Cs of PYD; and secondly, the profiles will be examined in relation to both anxiety and COVID-19 anxiety. It is anticipated that several different profiles will emerge (one with higher Five Cs, one with higher efficacious Cs, one with higher socioemotional Cs, and one with lower Five Cs). Further, gender and age will be included as the exploratory covariate variables, though, it is expected that more females will be included in the profile with socioemotional Cs and more males will be in the profile with efficacious Cs. Finally, we anticipate that the profiles characterized by high caring and high character will be associated with high anxiety and COVID-19 anxiety and the profiles with efficacious Cs will be associated with lower anxiety and lower COVID-19 anxiety. The present study presents a novel approach to exploring the association between both general and situation-specific anxiety and the Five Cs, utilizing a person-centered approach within the same analysis. This study builds upon previous research that has investigated the relationship between the Five Cs and anxiety and adds to the existing literature by identifying distinct profiles of adolescents who may be more susceptible to anxiety and situation-specific anxiety in stressful situations.

## Method

### Participants

The present study included 1,977 participants (57.4% females) aged from 13 to 19 (*M*_*age*_ = 15.34, *SD* = 1.19). The majority of the participants attended upper-secondary school (*n* = 1399; 70.8%), while the remaining 578 participants attended lower-secondary school (*n* = 578; 29.2%). Among the participants, 7% were born outside Slovenia and 10.5% had one parent who was born outside Slovenia. Participants reported their mothers’ or caregivers’ educational level which allows us to better understand their socioeconomic position. Approximately 51.1% reported that their mothers or caregivers had completed higher education or higher vocational education programs, while 34.2% indicated that their mothers or caregivers had attained upper-secondary education. Moreover, 2.8% of participants reported that their mothers or caregivers had completed compulsory basic education, while only 0.2% mentioned that their mothers or caregivers had not completed this level of education. Interestingly, 11.5% of participants were unsure about the educational attainment of their mothers or caregivers, and 0.3% reported not having a mother or a caregiver.

### Measures

*The Five Cs*. The short version of the PYD questionnaire was used to measure the Five Cs (Geldhof et al., 2014a). The scale consists of 34 items answered on a five-point Likert scale (with responses ranging from *1* = strongly disagree to *5* = strongly agree). The items measure the Five Cs: competence (6 items, e.g., “I have a lot of friends”); confidence (6 items, e.g., “I am happy with myself most of the time”); caring (6 items, e.g., “When I see someone being picked on, I feel sorry for them”); character (8 items, e.g., “I hardly ever do things I know I shouldn’t do”); and connection (8 items, e.g., “I feel my friends are good friends”). The questionnaire is psychometrically adequate (Geldhof et al., 2014a). The reliability coefficients in the current study are as follows: 0.73 (competence); 0.92 (confidence); 0.87 (caring); 0.73 (character); 0.81 (connection). CFA (confirmatory factor analysis) was conducted, in which latent factors were allowed to correlate. After eight covariances among item errors (justified by content) were added, the CFA confirmed an adequate fit of the five-factor structure: χ^2^ (507) = 3595.25, *p* < 0.001, RMSEA (root mean square error of approximation) = 0.055, 90% CI [0.054, 0.057], CFI (comparative fit index) = 0.905; SRMR (standardized root mean squared residual) = 0.066.

#### Anxiety

To measure general anxiety, the Anxiety Scale for Children and Adolescents was applied (Lestvica anksioznosti za otroke in mladostnike; Kozina, 2012). This consists of 14 items (e.g., “I worry a lot”). The participants indicated how often the statements had been true for them during the last month on a Likert scale (*1* = never to *5* = always). The reliability and validity of the instrument were well-documented for Slovenian students (Kozina, 2012). Cronbach’s α in our study was 0.91. CFA confirmed an adequate fit of one factor after we added Five covariances among item errors that were justified by content: RMSEA = 0.080, 90% CI [0.075, 0.084], CFI = 0.936; SRMR = 0.040.

#### COVID-19 anxiety

A measure for COVID-19 anxiety (Kozina et al., 2021b) that was designed to measure specific situational anxiety during the pandemic was used. Three items from this scale were included (“I am worried about getting infected with COVID-19”, “I am worried about infecting others with COVID-19.”, I am worried that I or someone close to me will become seriously ill or die from COVID-19.”) and another three items were added (i.e., “I am worried that I won’t be able to hang out with my friends due to COVID-19,” “I am worried about contradictory information about COVID-19,” and “I am worried about social changes that are happening because of COVID-19 (e.g., less contact with others, more restrictions)”) to capture how the students perceived the social changes that were consequences of the pandemic, such as social isolation, school closures, etc. The participants indicated how often the statements were true for them during the COVID-19 pandemic (*1* = never to *5* = always). Cronbach’s α in our study was 0.81. CFA confirmed a good fit of one factor after three covariances among item errors were added (between three items that were added to the scale): RMSEA = 0.073, 90% CI [0.058, 0.089], CFI = 0.985; SRMR = 0.021.

### Procedure

The data were collected during the research project *Positive Youth Development in Slovenia: Developmental Pathways in the Context of Migration*. The target population consisted of students in the last grade of the lower-secondary level and at the upper-secondary level in Slovenia. For the data to reflect the population as closely as possible the sample from all the available types of upper-secondary schools in Slovenia was examined according to the percentage of students attending each type of school. The sampling design was adapted to project’s objectives, including both schools with a higher percentage of migrant students (target sampling) and schools that were invited regardless of the migrant status of their students (random sampling). In the present study, 21 lower-secondary schools and 19 upper-secondary schools agreed to participate. The questionnaires were administered in the Slovene language and the PYD questionnaire was translated before the beginning of the data collection by using a committee approach. Additional flexibility due to the pandemic and possible school closures was provided for the response format as schools decided if their students would participate online on or paper, with 58.83% of the students responding online and 41.17% on paper. After obtaining informed consent from their parents, the data collection took place in schools (two weeks before school closures) or at home during online school hours. Participants were asked which gender they identified with. The time was not limited and the participants were supervised by the school coordinator. It is important to consider the changes in the COVID-19 situation during data collection. At the beginning of data collection, schools were still opened, however, students needed to wear masks during school hours. During the data collection, schools closed and remote schooling was adopted in addition to several restrictive measures (e.g., movement restrictions outside of regions and municipalities, and limitations on outdoor gatherings).

### Data Analysis

Statistical analyses were performed using IBM SPSS Statistics 28.0 and Mplus 8.6. Firstly, we checked the data for the number and patterns of missing values, performed normality tests by calculating skewness and kurtosis, and looked at potential outliers. There were less than 1.4% missing values on the item level. Little’s MCAR test showed that missing values were not missing completely at random: χ^2^ (2570) = 2761.795, *p* = 0.004. However, the normed chi-square was acceptable (χ^2^ / *df* = 1.07), which indicates that the data were likely missing at random. Therefore, maximum likelihood with robust standard errors was used in the Mplus to handle missing data. Skewness values varied between −1.641 and 0.794 and kurtosis values varied between −1.119 and 3.223. There were no particular issues regarding normality or outliers.

Secondly, as our data were nested (1,977 students nested within 137 classes in 40 schools), we calculated intraclass correlation coefficients (ICCs). ICCs demonstrate the shares of variance at each level. The ICCs in the present study were low as they ranged between 0.00 and 0.05 for schools and between 0.01 and 0.05 for classes, except for connection (ICC_class_ = 0.07). Given that the majority of the ICCs were lower or at the suggested cutoff of 0.05 (LeBreton & Senter, 2008), with one ICC being lower than 0.10 which is still considered as an acceptable value for the individual-level analyses (Peugh, 2010) and regarding that the aims of the present study correspond to individual characteristics, we decided to perform the analyses on the individual level.

After the descriptive statistics and correlations were examined, CFA and latent profile analysis (LPA) were performed using Mplus Version 8.6 (Muthén & Muthén, 1998–2021). LPA with maximum likelihood with robust standard errors as the estimator was used to recognize latent subgroups of participants based on the 5Cs (Nylund-Gibson & Choi, 2018). The modeling process started by estimating a one-profile LPA model; afterward, the number of profiles was increased while considering how adding another profile affected the fit indices, which are the guidelines used when deciding upon the number of profiles (Nylund-Gibson & Choi, 2018). The following fit indices were considered: information criteria, which include the Bayesian Information Criterion (BIC), where the profile solution with the lowest value for the information criteria, and a likelihood-based test: the Vuong–Lo–Mendell–Rubin adjusted likelihood ratio test (VLMR-LRT) were preferred. In addition, entropy as an index of the classification of individuals into profiles (values > 0.80) and average posterior probabilities as an index of the good separation of individuals into their most likely profile (AvePP; values > 0.70) as guidelines for the assessment of profile differentiation (Masyn, 2013) were examined. Furthermore, profiles were compared in IBM SPSS Statistics 28.0 software where Welch’s ANOVA and post-hoc tests (Games-Howell correction) were employed as the data had unequal variances.

Lastly, a multinomial logistic regression was employed to assess gender and age differences among profiles and the Bolck–Croon–Hagenaars (BCH) approach was used to examine the differences between anxiety and COVID-19 anxiety across the latent profiles (Asparouhov & Muthén, 2014). The BCH approach is recommended for continuous variables as it avoids profile changes and is susceptible to the differences in the variance of auxiliary variables across latent profiles (Asparouhov & Muthén, 2014).

## Results

### Descriptive Statistics

Prior to the analyses, the latent factor scores were computed (all means were centered to zero) and, after that, the means, standard deviations, and correlations were examined (see Table 1). All of the Five Cs except for confidence and caring were positively correlated, with correlation coefficients ranging from 0.11 to 0.62, meaning higher levels of competence, confidence, character, caring, and confidence. Competence, confidence, and connection were negatively correlated with gender, while character and caring were positively correlated with gender: males reported higher competence, confidence, and connection and lower character and caring than females. Out of the Five Cs, only competence and connection were negatively correlated with age, suggesting that older participants reported lower competence and connection. All of the Five Cs were correlated with anxiety. Competence, confidence, and connection were negatively correlated, meaning that higher levels of anxiety correspond to lower levels of competence, confidence, and caring. On the other hand, character and caring were positively correlated with anxiety, indicating that participants with higher character and caring had higher anxiety. As for COVID-19 anxiety, only competence was negatively correlated with this construct, suggesting that participants with higher levels of competence had lower levels of COVID-19 anxiety. Contrarily, character, caring, and connection were positively correlated with COVID-19 anxiety, meaning that those with higher COVID-19 anxiety had higher character, caring, and connection.

**Table 1:** Descriptive Statistics and Correlations of the Whole Sample

	*M*	*SD*	1.	2.	3.	4.	5.	6.	7.	8.
1. Gender										
2. Age	15.34	1.19	0.01							
3. Competence	3.43	0.68	−0.24***	−0.09***						
4. Confidence	3.56	0.92	−0.26***	−0.02	0.62***					
5. Character	3.88	0.56	0.20***	−0.02	0.26***	0.22***				
6. Caring	4.01	0.76	0.31***	−0.02	0.11***	0.03	0.61***			
7. Connection	3.75	0.65	−0.05*	−0.11***	0.55***	0.58***	0.40***	0.30***		
8. Anxiety	2.76	0.81	0.35***	0.04	−0.35***	−0.47***	0.09***	0.22***	−0.28***	
9. COVID-19 anxiety	2.99	0.96	0.29***	0.10***	0.00	−0.07***	0.23***	0.30***	0.10***	0.44***

*p < 0.05; ***p < 0.001

### Profile Identification

First, the model fit indices (BIC and VLMR-LRT) for one- to Five-profile solutions (see Table 2 for the model fit indices and probability test) were compared. The model fit indices (i.e., VLMR-LRT) revealed that the best solution was a four-profile solution. BIC was constantly decreasing, which can happen in LPA when larger samples are used (Marsh et al., 2009), but VLMR-LRT was significant for a four-profile solution. In the meantime, profile counts and proportions were considered as well. One of the profiles in the four-profile solution consisted of approximately 9% of the whole sample, while in the Five-profile solution, one of the profiles included only 3% of the whole sample, indicating that this profile solution may be over-extracted (Nylund-Gibson & Choi, 2018). In addition, the average posterior probabilities were examined, which were around 0.90 for each profile, showing that the profiles were well separated. Furthermore, entropy was considered, and suggested that the profiles were well differentiated (entropy > 0.80). The participants were divided into profiles according to the degree of the Five Cs, confirming that the four-profile solution was a suitable choice.

**Table 2:** Latent Profile Fit Statistics, Entropy, and the Highest Average Posterior Probabilities for the Five Cs with Covariates (fit statistics without covariates are in brackets)

P	BIC	VLMR-LRT (*p*-value)	Entropy	AvePP
1	24999.71 (15197.00)	/	/	
2	12856.29 (12177.67)	<0.001 (<0.001)	0.84 (0.85)	0.93 (0.93)
3	11183.12 (10304.56)	<0.001 (<0.001)	0.86 (0.87)	0.87 (0.92)
4	10320.32 (9283.66)	<0.001 (<0.001)	0.84 (0.88)	0.89 (0.93)
5	9666.53 (8675.13)	0.280 (0.366)	0.86 (0.89)	0.95 (0.97)

*P* profiles, *BIC* Bayesian information criterion, *SABIC* sample-size adjusted Bayesian information criterion, *VLMR-LRT* Vuong–Lo–Mendell–Rubin adjusted likelihood ratio test, *AvePP* average posterior probability

As a result of the significant correlations of the Five Cs with age and gender, both variables were subsequently added to the model (Nylund-Gibson & Masyn, 2016). The model fit indices are presented in Table 2 and confirmed that a four-profile solution was the optimal one. Adding the covariates to the model changed the distribution of the participants, as they were no longer divided according to the degree of the Five Cs. The profile division after adding the covariates was dependent on the individual Cs and not on all of the Five Cs as before. The profile counts, entropy, and AvePP remained adequate.

**Fig. 1:**
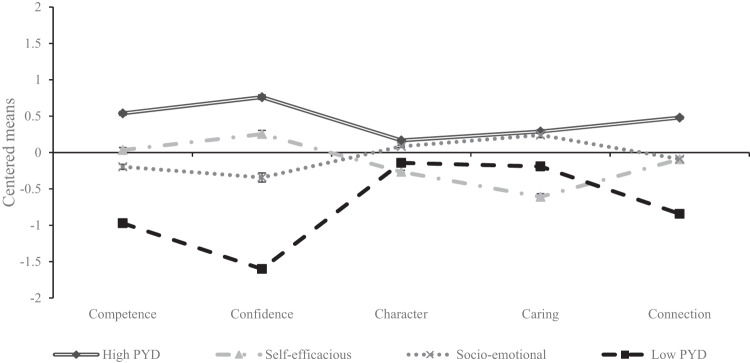
Mean Scores of the Five Cs in Identified Profiles with Covariates

The final profile classification is presented in Fig. 1. The distribution showed that the profiles differed on all of the Five Cs. The first profile, named “High PYD” (*n* = 657; 33.23%), contained students who reported the highest Five Cs scores among all profiles. The second profile, named “Self-efficacious” (*n* = 467; 23.62%), included students whose competence and confidence were higher, whose connection was moderate, and whose caring and character were the lowest among all the profiles. The third profile, named “Socio-emotional” (*n* = 592; 29.94%), consisted of students who reported higher caring and character, while they reported lower competence and confidence and moderate connection. The fourth profile, named “Low PYD” (*n* = 261; 13.20%), included students who reported the lowest competence, confidence, and connection, while they reported moderate caring and character.

All profiles quantitatively differed in all of the Five Cs as presented in Table 3. Welch’s ANOVA was used to assess differences across profiles since the data exhibited unequal variances. Almost all post-hoc comparisons were significant at the *p* < 0.001 level, except for High PYD and Socio-emotional profiles that did not differ in caring (*p* = 0.552) and Socio-emotional and Self-efficacious profiles that did not differ in connection (*p* = 0.994). According to Cohen (1988), all partial eta squared (η^2^) effect sizes were large.

**Table 3:** Means and Standard Errors of the Five Cs across Latent Subgroups and Comparisons Between Them

	High PYD	Self-efficacious	Socio-emotional	Low PYD	*F*	η^2^
*n* (%)	657 (33.23%)	467 (23.62%)	592 (29.94%)	261 (13.20%)		
Competence^a^	0.54 (0.03)	0.04 (0.03)	−0.20 (0.04)	−0.97 (0.05)	1376.14***	0.723
Confidence^b^	0.76 (0.03)	0.25 (0.05)	−0.34 (0.06)	−1.60 (0.17)	1394.55***	0.701
Character^c^	0.17 (0.01)	−0.27 (0.02)	0.09 (0.02)	−0.14 (0.03)	480.89***	0.411
Caring^d^	0.29 (0.03)	−0.61 (0.04)	0.24 (0.03)	−0.19 (0.06)	472.62***	0.404
Connection^e^	0.48 (0.02)	−0.09 (0.03)	−0.09 (0.03)	−0.84 (0.04)	1617.60***	0.749

*n* = number of participants in each profile. ***p < 0.001. Welch’s ANOVA was used since the data demonstrated unequal variances:

^a^*F*(3, 845.79)

^b^*F*(3, 847.61)

^c^*F*(3, 817.70)

^d^*F*(3, 817.27)

^e^*F*(3, 842.87)

Moreover, a four-profile solution was not only empirically viable but also theoretically justified as it captured the heterogeneity in the distribution of each C within individuals. The latent profile analysis revealed distinct profiles among participants: one characterized by elevated levels on all Five Cs, another marked by lower scores on most of the Five Cs (Johnson, 2021), a profile emphasizing socioemotional Cs with higher levels of caring and character, and a profile emphasizing efficacious Cs with greater competence and confidence (Årdal et al., 2018).

### Differences between Profiles in Gender, Age, Anxiety, and COVID-19 Anxiety

Age and gender were included as covariates and multinominal logistic regression was performed with the High PYD profile as the reference group. In regard to gender, all the profiles differed from the High PYD profile. The odds of being in the Self-efficacious profile (OR = 0.23, SE = 0.05, *p* < 0.001) were lower for girls, while the odds of being in the Socio-emotional profile (OR = 5.23, SE = 1.22, *p* < 0.001) or in the Low PYD profile (OR = 2.99, SE = 0.66, *p* < 0.001) were higher for girls compared to the High PYD profile. As for age, differences were revealed only for the Self-efficacious and Low PYD profiles in comparison with the High PYD profile. The odds of being in the Self-efficacious profile (OR = 1.14, SE = 0.07, *p* = 0.047) or in the Low PYD profile (OR = 1.18, SE = 0.08, *p* = 0.015) were higher for older students (see Table 4).

**Table 4:** Standardized Means and Standard Errors of Auxiliary Variables and Tests of Mean Differences across Subgroups with Covariates

	Anxiety	COVID-19 anxiety
*M* (*SE*)		
High PYD	−0.51 (0.04)	0.07 (0.04)
Self-efficacious	−0.51 (0.04)	−0.58 (0.04)
Socio-emotional	0.49 (0.04)	0.37 (0.04)
Low PYD	1.09 (0.06)	−0.02 (0.06)
Overall test (Wald χ^2^)^†^	916.95***	246.46***
Pairwise tests (Wald χ^2^)^‡^		
High PYD vs. Self-efficacious	0.00	115.86***
High PYD vs. Socio-emotional	377.61***	27.87***
High PYD vs. Low PYD	540.63***	1.72
Self-efficacious vs. Socio-emotional	348.80***	240.62***
Self-efficacious vs. Low PYD	524.07***	59.59***
Socio-emotional vs. Low PYD	71.62***	30.40***

***p < 0.001

^†^All tests have 1 degree of freedom

^‡^All tests have 3 degrees of freedom

**Fig. 2:**
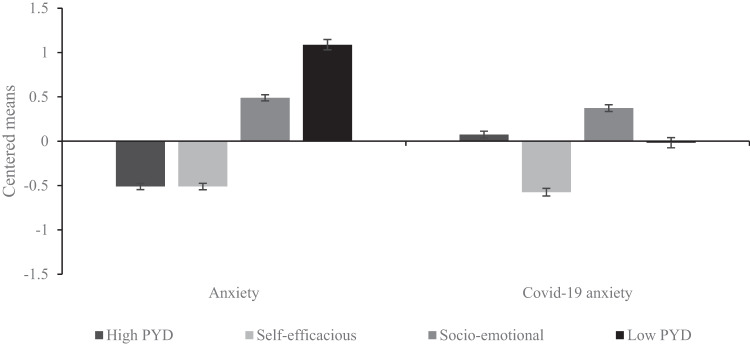
Comparison of Identified Profiles with Covariates in Anxiety and COVID-19 Anxiety

Differences between the latent subgroups were examined using the BCH approach. Figure 2 represents how the profiles differ for anxiety and COVID-19 anxiety. Wald’s chi-square test showed that students in the High PYD and Self-efficacious profiles reported the lowest anxiety and their anxiety scores did not differ from one another. Students in the Socio-emotional profile reported higher anxiety while participants in the Low PYD profile reported the highest anxiety among all the profiles. As regards COVID-19 anxiety, students in the Self-efficacious profile reported the lowest COVID-19 anxiety among all the profiles, while students in the High PYD and Low PYD profiles reported similar, moderate COVID-19 anxiety. Students in the Socio-emotional profile reported the highest COVID-19 anxiety.

## Discussion

The COVID-19 pandemic triggered mental health issues among adolescents, including anxiety and COVID-19 anxiety (Panchal et al., 2021). However, it is important to recognize that not all adolescents may be equally affected and some may require more targeted and urgent support through tailored prevention and intervention programs. One way of identifying these youth is by using a person-centered approach. The present study used the PYD framework and person-centered analysis to identify the patterns of positive outcomes among youth – that is, the Five Cs – to provide a scientific background for tailored prevention and intervention strategies. The data collection took place during the initial stages of the second closure in Slovenia, which involved prolonged school closures. Thus, this study presents a unique opportunity to observe youth’s adaptation mechanisms in an extremely stressful situation and can provide valuable implications for similar health and social isolation challenges in the future.

As anticipated, four distinctive profiles were found, namely High PYD profile, Self-efficacious profile, Socio-emotional profile, and Low PYD profile. The High PYD and Low PYD profiles are consistent with a previous study (Johnson, 2021) since a profile with high scores for all of the Five Cs and a profile with low scores for all of the Five Cs were identified in this study as well. Further, the Self-efficacious and Socio-emotional profiles are in line with a previous study (Årdal et al., 2018) that proposed that the Five Cs can be differentiated into efficacious Cs (i.e., competence, confidence, and connection) and socioemotional Cs (i.e., caring and character). In addition, a similar profile was shown (Johnson, 2021), named “midpoint with a caring emphasis”, which was characterized by average overall PYD, although their caring was higher. The profiles identified in this study bear similarity to those observed prior to the onset of the COVID-19 pandemic. However, a decrease in PYD constructs during this global crisis was documented (Wang et al., 2023). Therefore, it is possible that the distribution across the Five Cs may remain relatively stable and the levels of the Five Cs in each profile decreased due to the COVID-19 pandemic. Thus, these profiles may include a combination of stable individual characteristics and pandemic-specific influences on adolescents’ well-being. Additionally, the COVID-19 pandemic may have impacted some individuals more than others, therefore, some perceived greater changes in the Five Cs during the pandemic. However, it is important to note that the specific nature of these profiles in relation to the pandemic and their stability over time remain uncertain due to the cross-sectional study design.

To get a better insight into the characteristics of the profiles, the differences between the odds of being in the High PYD profile and the odds of being in the other profiles across the PYD profiles in gender and age were examined. Females had a higher chance than males of being in the Socio-emotional and Low PYD profiles, while males had a higher chance than females of being in the Self-efficacious profile. Our findings are in line with our expectations and previous research, which suggested that males are more likely to be present in profiles with higher levels of competence and confidence, such as the Self-efficacious profile in our study (Gomez‐Baya et al., 2019). Conversely, females are more likely to be present in profiles characterized by higher levels of caring, character, and connection, such as the Socio-emotional profile in our study (Årdal et al., 2018). It appears that higher levels of caring and character are the main characteristics that reflect differential male and female involvement across the different profiles, which can be explained by gender role expectations (e.g., Van der Graaff et al., 2014). Specifically, these gender role expectations may contribute to the development of socialization processes that shape individuals' attitudes and behaviors. Such expectations may differ across genders, with females being encouraged to prioritize caring, and males being encouraged to prioritize competence and confidence. Based on the findings and the notion that elevated levels of the Five Cs contribute to enhanced adaptive developmental processes, targeted interventions tailored to the unique profiles, differentiated by gender, can be implemented. Specifically, for males, interventions focusing on supporting caring may prove beneficial, while for females, interventions designed to cultivate competence and confidence may be particularly effective.

With respect to age differences, our analysis showed that older students had higher odds of being in the Self-efficacious and Low PYD profiles than in the High PYD profile. While age-related differences were not anticipated due to the homogeneity of the sample, our findings are partially in line with previous studies (Conway et al., 2015) that have reported higher levels of character, caring, connection, and overall PYD scores among younger adolescents. However, our results indicate that older participants in the Low PYD and Self-efficacious profiles reported lower levels of character and caring, while their levels of connection were either lower or average. This suggests that PYD, in general, may decline over adolescence (Conway et al., 2015), which is consistent with findings that competence, confidence, and connection tend to decrease over time (Geldhof et al., 2014b). Thus, specific prevention measures supporting all of the Five Cs with a particular emphasis on caring and character can be implemented for older students.

Once the profiles were identified, the differences between anxiety and COVID-19 anxiety among them were further investigated. The participants in the Low PYD profile seem to be the most at risk of general anxiety and possibly other general mental health issues. The combination of the lowest levels of competence, confidence, and connection alongside moderate levels of character and caring may contribute to excessive worrying. When individuals perceive that they lack necessary skills (i.e., in the school context, among peers), this increases feelings of uncertainty, which in turn leads to higher anxiety (Tahmassian & Moghadam, 2011). Additionally, lower perceived social support may contribute to social anxiety (Cavanaugh & Buehler, 2016). Moreover, participants in the Low PYD profile may be at risk of adopting maladaptive developmental regulations (Geldhof et al., 2019), meaning that their actions may be detrimental to themselves and their context. For instance, they may use maladaptive coping strategies and risky behavior to please others and gain their attention (e.g., substance abuse, school avoidance) or avoid situations that trigger anxiety. Interestingly, participants in the Low PYD profile had moderate COVID-19 anxiety, which could be attributed to their lower level of connection with others and moderate caring. Thus, the lack of social ties and support combined with a moderate level of concern and empathy about significant others indicate a certain degree of COVID-19 anxiety but not to an excessive extent. Overall, this profile is the most at risk due to heightened levels of anxiety which may persist even after the COVID-19 pandemic.

The Socio-emotional profile, in comparison to all other profiles, reported the highest levels of COVID-19 anxiety and moderate anxiety. It consisted of participants with higher character and caring, moderate connection, and lower competence and confidence. It appears that the interaction between higher caring and lower competence and confidence is a key factor in COVID-19 anxiety across different profiles as prior research confirmed the maladaptive consequences of caring (Kozina et al., 2021a) and the profile with higher caring and character scores reported more somatic complaints (Ferrer-Wreder et al., 2021). These findings suggest that anxiety is positively associated with caring and offer valuable insights into understanding the relationship between anxiety and caring. Furthermore, a recent study showed that higher trait anxiety is associated with higher empathy during the COVID-19 pandemic (Guadagni et al., 2020). Elevated levels of anxiety can be a result of a complex interplay between lower competence and higher caring that can be seen as a lack of metacognitive awareness (Kozina et al., 2021a), which allows one to distinguish between one’s emotional state and that of others, thus, increasing the chance of emotional contagion or empathic over-arousal to occur (Hoffman, 2008). Additionally, the Socio-emotional profile may experience martyring developmental regulations (Geldhof et al., 2019) as their heightened caring often leads to worrying about the welfare and safety of others, which can negatively impact themselves while others or their context may benefit from them. The burden of prevention measures for COVID-19 – the constant media messages showing the severe course of the disease (e.g., overcrowded hospitals, full morgues), and clear government messages stating that we are all a threat to other people and that we have to follow preventive measures to protect the most vulnerable groups, otherwise they will die – has been particularly harmful. For individuals in the Socio-emotional profile, who are often more empathetic and sympathetic (Panchal et al., 2021), this was a "lethal" combination. Regarding contribution, which is a fundamental need of adolescents (Fuligni, 2019) and is embedded in the PYD perspective as a positive outcome of the Five Cs (Lerner et al., 2015), it was found that prosocial acts during the COVID-19 pandemic were connected to greater anxiety (Alvis et al., 2022). It is possible that individuals who perceived greater COVID-19 anxiety sought additional ways to help others with similar experiences. Conversely, contribution to others during the COVID-19 pandemic alleviated their anxiety (Alvis et al., 2022).

The participants in the Self-efficacious profile exhibited higher levels of competence and confidence while they had the lowest caring and character among all profiles, as well as having the lowest anxiety and COVID-19 anxiety. Higher levels of competence and confidence may offer a possible explanation for these phenomena since both characteristics were found to correlate negatively with anxiety (e.g., Mazzone et al., 2007; Soleimani et al., 2017). These results further support our finding that the Self-efficacious profile has the lowest levels of COVID-19 anxiety. A study based on a sample of emerging adults during the COVID-19 pandemic (Germani et al., 2020) showed that participants in the group with low anxiety had the highest levels of self-esteem and self-efficacy, suggesting that these participants may have been more resilient to the challenges society was facing at that time. Additionally, higher levels of confidence have been shown to help individuals to cope more successfully with day-to-day challenges (Soleimani et al., 2017). High competence and confidence are undeniably valuable assets that help participants in this group to cope better. However, this group should be given additional attention as the interaction of high competence and confidence with low levels of character and caring may make participants in this profile less likely to engage in prosocial behavior (e.g. Geldhof et al., 2019). During the COVID-19 pandemic in particular, individuals with similar characteristics were less likely to engage in healthy behaviors and tended to continue living as if nothing had happened (Triberti et al., 2021).

Participants in the High PYD profile had the highest Five Cs, the lowest anxiety, and moderate COVID-19 anxiety. This suggests that higher competence, confidence, and connection can buffer the maladaptive effect of higher caring on anxiety. These findings highlight the importance of supporting all of the Five Cs to prevent youth from adverse consequences. However, the findings about moderate COVID-19 anxiety suggest that it is also important to assess situation-specific anxiety in times of extremely stressful situations to be able to offer additional support even to those who are at the lowest risk of general anxiety. It seems that the current COVID-19 situation has affected a large proportion of adolescents, especially those with higher caring. However, participants in the High PYD profile had moderate COVID-19 anxiety, so besides competence and confidence, connection might have protected them from having even higher COVID-19 anxiety. This confirms a finding that friendship satisfaction at the start of the first lockdown was a negative predictor of anxiety one month later (Stevic et al., 2022). Participants in the High PYD profile with higher scores on all Five Cs experience adaptive developmental regulations (Lerner et al., 2005) and may have higher contribution than youth in other profiles (Johnson & Ettekal, 2023). However, during the COVID-19 pandemic, their higher caring has led to increased worrying about their significant others and generated COVID-19 anxiety (Panchal et al., 2021). This suggests that even the most well-adapted adolescents can experience martyring developmental regulations during extreme crises, potentially negatively impacting their mental health. Thus, it is essential to prioritize the mental health of all adolescents during future crises.

### Implications for Practice

The findings of the present study have practical implications for alleviating anxiety and COVID-19 anxiety among adolescents. The results indicate that particularly higher levels of competence and confidence can mitigate the maladaptive effect of higher caring in the case of general anxiety. Hence, it is crucial to create supportive environments that promote positive developmental trajectories for all students. Such support can facilitate the adaptive developmental regulations (Geldhof et al., 2019) and equip them to overcome possible future challenges, even in High PYD and Self-efficacious profiles, which had low or moderate levels of anxiety and COVID-19 anxiety. Furthermore, given the potential for maladaptive developmental outcomes for profiles with the highest anxiety and COVID-19 anxiety, additional attention and support are needed. Participants in the Socio-emotional profile with martyring developmental regulations should benefit from incorporating social and emotional learning in school with a particular focus on self-awareness (i.e., identifying positive beliefs about oneself and one’s achievements and recognizing one’s potential), self-management, and relationship skills. Prevention and intervention measures should focus in particular on helping them differentiate their emotional states from those of their significant others, especially during periods of heightened stress such as the COVID-19 pandemic. Emphasizing social belonging (to achieve higher connection) is also crucial (Slavich et al., 2021), and introducing relaxation techniques may help manage the negative effects of the pandemic. Participants in the Low PYD profile should particularly benefit from interventions that target competence, confidence, and connection. Techniques from cognitive behavioral therapy, such as cognitive restructuring, relaxation, stress reduction techniques, identification of negative or distorted thoughts, thought stopping, and problem-solving, may be useful. Additionally, support groups, social skills training, and providing additional support to improve their relationships with peers, parents, and teachers can boost their connection with others.

### Limitations and Implications for Future Studies

One of the notable strengths of our study is that it is one of the first to examine PYD profiles based on the Five Cs, providing novel insights into the relationship between these profiles and anxiety, including COVID-19 anxiety. By incorporating situation-specific anxiety into the PYD perspective, our study extends previous research examining the complex relationship between the Five Cs and anxiety, particularly with regard to caring (e.g., Geldhof et al., 2019). Further, our data were collected during a period of heightened stress, amidst the second wave of the COVID-19 pandemic and school closures in Slovenia.

Notwithstanding the strengths of our study, it is important to acknowledge several limitations. The response format was adapted to COVID-19 restrictions (online and paper-pencil forms were used) and the COVID-19 pandemic affected the rate of responses due to school closures. Furthermore, our reliance solely on self-reports may have introduced bias and influenced the accuracy of the results. Nevertheless, the study aimed to capture adolescents’ current experiences during the COVID-19 pandemic. Additionally, a cross-sectional research design was utilized, thus we do not know if the profiles are situation-specific or stable. However, using a cross-sectional research design allowed us to capture better insight into specific stressful situations since the context did not change as rapidly as it does when a longitudinal research design is employed. Another limitation is the exclusion of the contribution which was made based on the primary focus of our study that builds on the established empirical association between the Five Cs and anxiety. Lastly, while our study included students with a migrant background, this characteristic was not specifically addressed in our analysis. However, it is essential to recognize that individuals with a migrant background may have experienced unique challenges and adversities during the COVID-19 pandemic (e.g., language constraints).

In order to get an answer on the stability of the profiles and their dependence on the COVID-19 context, it makes sense that future studies employ longitudinal designs, such as latent transition analysis, to explore the changes in the PYD profiles over time, or growth mixture modeling to obtain a more normative picture of the PYD. Further, considering contribution as one of the Six Cs in profile identification would provide more insight into youth development. In addition, migration status and socioeconomic position can also be examined to provide more information on the challenges faced by youth from different backgrounds. Moreover, other characteristics (e.g., internalizing and externalizing behaviors) should be included in the examination of differences among the PYD profiles to capture a more comprehensive picture of the relationships between the Five Cs and various outcomes.

## Conclusion

Despite the well-documented rise in anxiety and COVID-19 anxiety during the COVID-19 pandemic, the supportive mechanisms from the PYD perspective have not yet been identified. To address this gap, the present study employed latent profile analysis to identify distinctive profiles based on the Five Cs, which were subsequently compared in anxiety and COVID-19 anxiety. Four distinct profiles were identified, illustrating the diverse nature of youth development: High PYD (high scores on all Five Cs), Self-efficacious (higher competence and confidence, lower character and caring, moderate connection), Socio-emotional (lower competence and confidence, higher character and caring, moderate connection), and Low PYD (lowest competence, confidence, and connection, moderate character and caring). These profiles exhibited distinct patterns in anxiety and COVID-19 anxiety, with the Low PYD profile showing the lowest anxiety and moderate COVID-19 anxiety, the Self-efficacious profile demonstrating the lowest anxiety and COVID-19 anxiety, the High PYD profile presenting the lowest anxiety and moderate COVID-19 anxiety, and the Socio-emotional profile displaying moderate anxiety and the highest COVID-19 anxiety. These findings suggest that higher levels of competence and confidence can serve as buffers against the negative effects of caring on anxiety, thereby providing a starting point for targeted interventions. Interestingly, the profiles exhibited distinct patterns of response in relation to COVID-19 anxiety, highlighting the complex interplay between the Five Cs and the unique challenges posed by the pandemic. This suggests that a combination of higher competence and confidence, along with lower caring, may contribute to a more adaptive response to the anxiety-inducing aspects of the COVID-19 situation. Overall, these findings significantly advance our understanding of adolescence by emphasizing the importance of considering the Five Cs and their intricate interplay with general anxiety and COVID-19 anxiety. By identifying distinct profiles and elucidating their associations with anxiety outcomes, this research provides valuable insights that can inform the development of interventions and support efforts aimed at promoting PYD and mitigating anxiety among adolescents, particularly during challenging times such as the COVID-19 pandemic.
